# Comparison of Anthropometric Parameters after Ultralow Anterior Resection and Abdominoperineal Resection in Very Low-Lying Rectal Cancers

**DOI:** 10.1155/2018/9274618

**Published:** 2018-06-10

**Authors:** Jun Woo Bong, Seok-Byung Lim, Jong Lyul Lee, Chan Wook Kim, Yong Sik Yoon, In Ja Park, Chang Sik Yu, Jin Cheon Kim

**Affiliations:** Department of Surgery, Asan Medical Center, University of Ulsan College of Medicine, 88 Olympic-ro 43-gil, Songpa-gu, Seoul 05505, Republic of Korea

## Abstract

**Background and Aim:**

Ultralow anterior resection (uLAR) is a sphincter-saving procedure for very low-lying rectal cancers. This procedure, however, has complications related to defecation which can aggravate the patient's quality of life postoperatively. In this study, we compared the anthropometric and nutritional parameters after uLAR and abdominoperineal resection (APR).

**Methods:**

We retrospectively reviewed the data of patients who underwent either uLAR or APR in 2012 for rectal cancers within 3 cm from the anal verge. Data including body weight, body mass index (BMI), levels of total protein, albumin, and hemoglobin and lymphocyte count were analyzed. We compared the changes of these parameters before operations to 3 years after discharge between uLAR and APR groups by ANOVA for repeated measures and Bonferroni comparison method.

**Results:**

After 3 years of discharge, the body weight and BMI of the APR group were fully recovered to the preoperative levels; however, those of the uLAR group did not. The hemoglobin level in the APR group was recovered to the preoperative level within 3 months of discharge; however, that in the uLAR group was recovered after 1 year of discharge.

**Conclusions:**

Recovery of anthropometric and nutritional status of patients was more stable after APR than after uLAR. These findings might indirectly reflect the low anterior syndrome effect of uLAR and help colorectal surgeons in selecting better surgical methods and in better counseling patients with very low-lying rectal cancer.

## 1. Introduction

The main goals in the surgical treatment of rectal cancer are complete resection with total mesorectal excision and conservation of the sphincter function. However, in cases in which a tumor-free distal resection margin is not achievable, a locally far-advanced tumor is present, and the patient shows severely decreased anal function preoperatively, surgeons should perform abdominoperineal resection (APR) requiring a permanent colostomy. Permanent colostomy can lead to psychological problems. Colostomy was reported to be associated with depression, low self-esteem, and low rates of social participation [1]. However, there is controversy about the patients' quality of life (QoL) with a permanent colostomy. Some authors reported that patients undergoing APR tended to show better physical, emotional, and social function and reported less fatigue and gastrointestinal symptoms than patients undergoing low anterior resection [2, 3].

Ultralow anterior resection (uLAR), as a sphincter-saving procedure, has been developed for the treatment of very low-lying rectal cancers. Recently, surgeons have been encouraged to perform uLAR with a better understanding of the distal surgical margin, adaptation of preoperative chemoradiation therapy (PCRT), and development of surgical techniques such as intersphincteric resection [4, 5]. However, the lower the anastomotic level after uLAR, the greater the likelihood of functional disorder development, the so-called low anterior resection syndrome (LARS) [6, 7]. LARS includes symptoms of incontinence for flatus, urgency, and frequent bowel movements and has been associated with a negative impact on the QoL [8, 9]. The main causes of LARS are poor function of the neorectum, sphincter damage, and surgical denervation of the rectum or anal sphincter [10].

In general, patients complain of sleeplessness, diarrhea, and difficulty in controlling stools and eventual loss of appetite with decreased body weight at the outpatient clinic during the follow-up period after uLAR. However, there is a paucity of studies on the effects on anthropometric status, including body weight change of LARS, after uLAR. Therefore, the main objective of the present study was to compare the postoperative changes in the patients' anthropometric and nutritional status after APR and uLAR for the treatment of low rectal cancer and to identify the factors predicting the clinical and nutritional changes postoperatively.

## 2. Materials and Methods

### 2.1. Patients and Data

**Figure 1:**
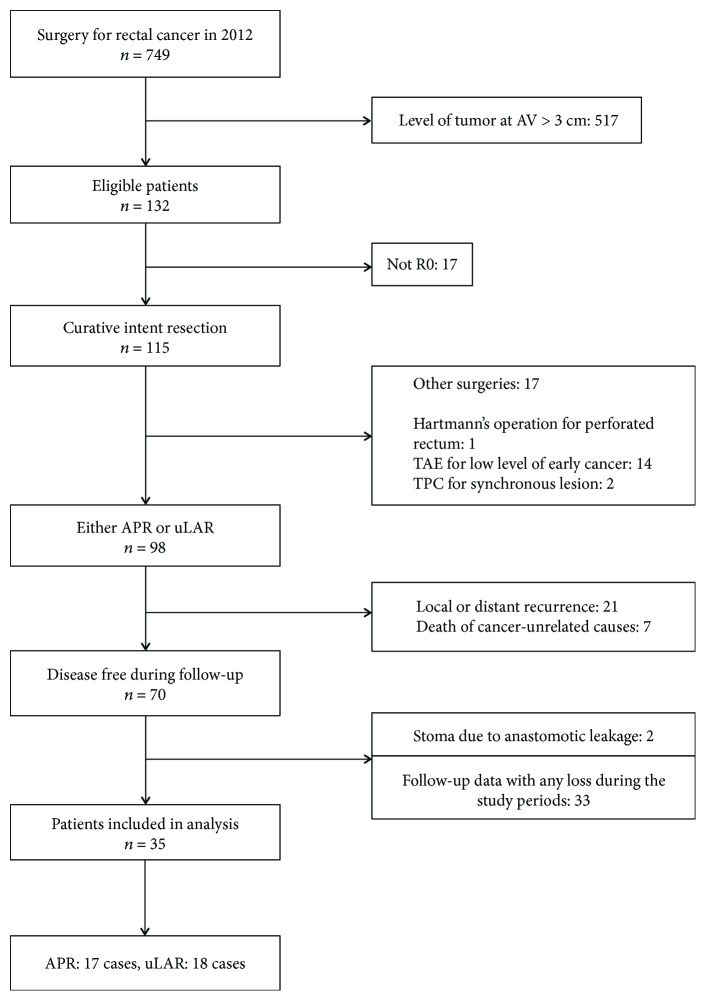
Flowchart of patient selection. AV: anal verge; APR: abdominoperineal resection; uLAR: ultralow anterior resection; TAE: transanal excision; TPC: total proctocolectomy.

A total of 749 patients underwent surgery for rectal cancer from January to December 2012 at Asan Medical Center and had a minimum follow-up of 36 months. The data were extracted from a prospectively collected colorectal cancer registry in our division of colorectal surgery and analyzed retrospectively. Of the 749 patients, 132 had a very low-lying rectal cancer (≤3 cm from the anal verge). The inclusion criteria are shown in Figure 1. Finally, 35 patients were included in our study and were divided into two groups according to the type of operation they underwent for rectal cancer: 17 patients were categorized into the APR group and 18 patients into the uLAR group.

We reviewed and compared the data of each group. The preoperative variables were age at operation, sex, PCRT, location of tumor from the anal verge, clinical T/N categories, and time interval from PCRT to the operation. Operative factors such as method of ligation of the inferior mesenteric artery (high versus low ligation) and surgical approach (open versus minimally invasive) and pathologic findings including histological differentiation and pathological T/N categories were reviewed. Whether patients received adjuvant chemotherapy or radiotherapy was also recorded.

Body weight and hematological/biochemical parameters were investigated to assess the nutritional status of the patients. The hematological/biochemical parameters included the levels of total protein, albumin, and hemoglobin and the lymphocyte count. Preoperative body weight was measured at the time of admission for surgery, and body weight was measured again at discharge. Body mass index (BMI) was calculated using the standard formula: weight (kg)/height (m^2^). When patients visited the outpatient clinic after discharge according to their regular follow-up schedules, all these parameters were measured. All parameters were assessed preoperatively; at discharge; at 1, 2, and 3 months after discharge; and at 1, 2, and 3 years after discharge. The changes in body weight and other nutritional parameters were measured by calculating the proportion of change at the designated time after discharge compared with the preoperative value (proportion of change = [(value at the designated time after discharge − preoperative value)/preoperative value] × 100%). To investigate the risk factors of weight loss at 3 years after discharge, we analyzed the clinicopathologic variables mentioned above. We defined weight loss as a weight reduction at 3 years after discharge compared with the preoperative weight. This study was approved by the Institutional Review Board of Asan Medical Center (IRB approval number: S2017-2246-0001).

### 2.2. Operation

All surgeries were performed by colorectal surgeons who have at least a 5-year experience in treating rectal cancer. All patients in both groups underwent total mesorectal excision including radical resection of the primary tumor and regional lymph nodes and preservation of autonomic nerves in the pelvis. The decision on performing APR or uLAR was made by the surgeon according to intraoperative findings such as distal resection margin and invasion of the sphincter muscle. End-to-end anastomosis was constructed in all patients in the uLAR group with either the hand-sewn (*n* = 6) or double-stapling method (*n* = 12). All patients in the uLAR group received a loop ileostomy for diversion, and ileostomy closure was performed at about 6 months (5.7 ± 1.1 months) after the operation.

### 2.3. Statistical Analysis

Data were analyzed using SPSS software version 21.0 (SPSS, Chicago, IL, USA). Discrete values such as sex, clinical stages, and surgical approaches were compared using Pearson's *χ*^2^ test. Student's *t*-test was used to compare continuous values such as age, weight, and biochemical parameters. Data are presented as mean ± standard deviation. Analysis of variance (ANOVA) for repeated measures analysis was used to compare the patterns of changes in the anthropometric and nutritional data at discharge between the two groups, 1, 2, and 3 months after discharge and 1, 2, and 3 years after discharge. Bonferroni post hoc analysis was performed to compare the changes of variables at each time between the two groups. Univariate and multivariate analyses were performed using a logistic regression model for risk factors of weight loss at 3 years after discharge. Statistical significance was set at *p* < 0.05.

## 3. Results

### 3.1. Clinicopathologic Characteristics of Patients

The demographic and preoperative characteristics did not differ between the two groups except for the tumor location from the anal verge (Table 1). The mean distance of the tumor from the anal verge was shorter in the APR group (*p* < 0.001). The proportion of patients who received PCRT was not different between the two groups: 16 (94.1%) in the APR group and 16 (88.9%) in the uLAR group. There was also no difference in clinical stages. Parameters related to baseline nutritional status such as body weight; BMI; protein, albumin, and hemoglobin levels; and lymphocyte count did not differ between the two groups. The operative and pathologic results of the two groups also showed no significant difference (Table 2). Most operations were performed through an open approach, 16 cases (94.1%) in the APR group and 13 cases (72.2%) in the uLAR group, with no significant difference. All patients in the APR group and 16 patients (88.9%) in the uLAR group received adjuvant chemotherapy.

### 3.2. Evaluation of Postoperative Nutritional Status

**Figure 2:**
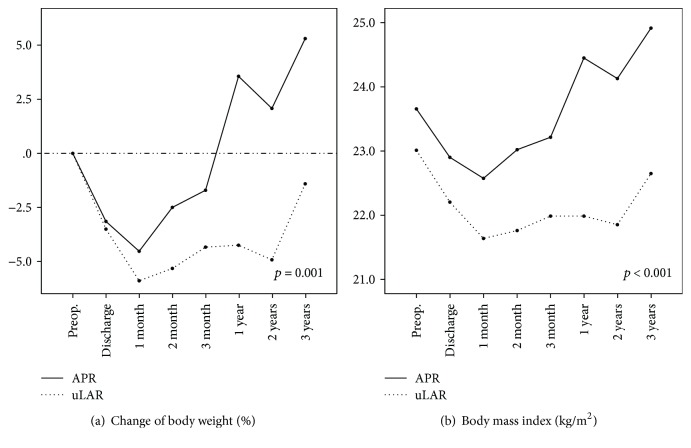
Changes in anthropometric parameters. Proportion of changed (a) body weight and (b) body mass index. APR: abdominoperineal resection; uLAR: ultralow anterior resection.

The analysis of body weight revealed that the patterns of body weight change at discharge; 1, 2, and 3 months after discharge; and 1, 2, and 3 years after discharge compared with the preoperative values had a significant difference between the two groups (*p* = 0.001, Figure 2(a)). The body weight change at 3 years after discharge was +5.30 ± 5.42% of the preoperative value in the APR group but −1.41 ± 7.31% in the uLAR group (*p* = 0.004). According to the results of Bonferroni comparison at each time, there were significant differences between the two groups in the weight changes at 1, 2, and 3 years after discharge (*p* < 0.001, *p* = 0.001, and *p* = 0.004, resp.). The patterns of BMI change during the same periods were also different between the two groups (*p* < 0.001, Figure 2(b)). The changes of BMI at 1, 2, and 3 years after discharge were also statistically different between the two groups after Bonferroni comparison at each time, (*p* < 0.001, *p* = 0.001, and *p* = 0.003, resp.). The BMI change at 3 years after discharge was +1.25 ± 1.30 kg/m^2^ of the preoperative BMI in the APR group but −0.36 ± 1.61 kg/m^2^ in the uLAR group (*p* = 0.003). The proportion of patients who experienced weight loss at 3 years compared with the preoperative weight was higher in the uLAR group: 13 patients (72.2%) in the uLAR group and 3 patients (17.6%) in the ARP group (*p* = 0.001). In univariate analysis, uLAR and a low tumor location from the anal verge were significant risk factors of weight loss after 3 years (*p* = 0.003 and 0.02, respectively; Table 3). uLAR was the only independent risk factor in multivariate analysis (*p* = 0.049).

**Figure 3:**
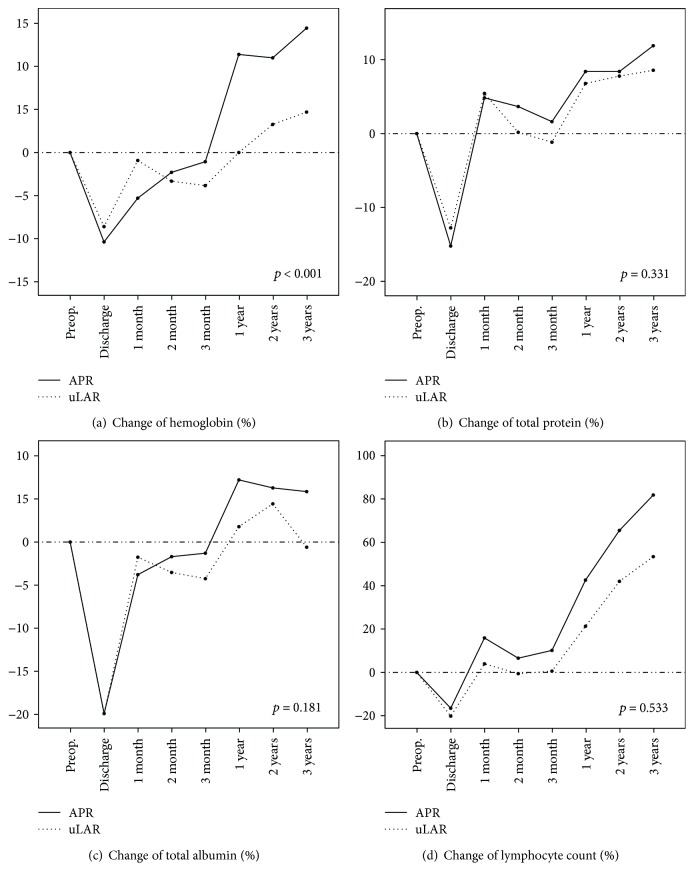
Changes in nutritional parameters. Proportion of changed (a) hemoglobin, (b) total protein, (c) total albumin, and (d) lymphocyte count. APR: abdominoperineal resection; uLAR: ultralow anterior resection.

Analysis of the hematologic and biochemical parameters showed that the pattern of changes in total hemoglobin level significantly differed during the same time intervals (*p* < 0.001, Figure 3(a)). There were significant differences between the two groups in hemoglobin changes at 1 and 3 years after discharge according to Bonferroni comparison at each time (*p* = 0.001 and *p* = 0.006, resp.). However, the changes in the levels of total protein and albumin and the lymphocyte count did not show a different pattern (Figures 3(b)–3(d)). Additionally, these variables showed no significant difference in changes at each time.

## 4. Discussion

With the acceptable oncologic outcomes and increased sphincter-saving rates, uLAR has recently replaced APR, and APR now tends to be performed in limited cases for the treatment of patients with rectal cancer. In our study, the uLAR group tended to recover their preoperative body weight more slowly than the APR group. Rather, the mean weight change at 3 years after discharge in the uLAR group was −1.41% of the preoperative value. Although we did not examine the changes of QoL of the two groups, we assumed that the difference in anthropometric and nutritional status might come from LARS. Many reports mentioned that patients with very low anastomoses are susceptible to the development of LARS, which could also severely impair the QoL, and recent studies showed the QoL after sphincter-saving surgery was not better than that after APR [11]. Konanz et al. [12] reported that uLAR also showed worse scores especially in appetite loss and weight loss than APR. Appetite and weight loss can result from LARS because many patients with this syndrome tend to worry about bowel habit changes or discomfort after eating. This can affect the postoperative nutritional status if the patient feels that consuming food is a burden and thus continuously avoids eating. Assessment of nutritional status is necessary in the long-term care of patients, and careful monitoring of nutritional status leads to an individualized plan of care. Patients undergoing major gastrointestinal surgery are frequently at a risk of developing malnutrition, not only due to the disease itself but also to the treatment processes and postoperative functional deterioration of the gastrointestinal tract [13]. The risk of malnutrition remains after surgery if postoperative gastrointestinal problems and limitation of dietary intake continue, which can also influence the QoL.

Generally, weight loss is the most prominent outcome during the period from 4 to 12 weeks after gastrointestinal tract surgeries [14]. However, the time to reach the preoperative weight mostly depends on how long it takes for patients to recover their preoperative QoL with a normal functioning body [13]. In this study, weight change was measured at monthly intervals until 12 weeks after discharge to evaluate the immediate body weight changes and at 1-year interval until 3 years after discharge to analyze the long-term weight changes. According to the ANOVA analysis, the body weights of patients in the APR and uLAR groups changed differently during the 3 years after discharge (*p* = 0.001). Patients in the APR group tended to recover their preoperative weight by 1 year after discharge, and their body weights seemed to continue to increase thereafter. However, it was difficult to conclude that the uLAR group showed a prominent recovery of body weight until 1 year after discharge. Their body weight seemed to have begun to increase in the third year, although their preoperative weight has not been recovered on average. Additionally, although the statistical difference was marginal (*p* = 0.049), uLAR was the only risk factor for weight loss at 3 years after discharge in the multivariate analysis.

The slow weight recovery rate of uLAR patients until 3 months after surgery may be attributable to the effect of ileostomy. Most nutrients are absorbed by the duodenum and jejunum; however, the ileum also absorbs a significant amount of nutrients including vitamins and electrolytes. As the bowel transit time of food is shortened in patients with an ileostomy, the absorption capacity of these patients can be decreased [15]. However, considering that all patients in our study underwent ileostomy closure at 6 months after surgery, ileostomy cannot be considered a factor affecting the weight change 1 year later. As there was no significant difference in the clinical courses between the two groups after 1 year of discharge, it could be inferred that the difference in body weight change between the two groups was due to LARS-induced changes in dietary habits. The change of body weight in the uLAR group was, however, not a clinicallysignificant involuntary weight loss, which is defined as a loss of 4.5 kg or >5% of the usual body weight during a period of 6–12 months [16]. Moreover, there was no statistical difference between the two groups in the change of total protein and albumin levels and the total lymphocyte count during 3 years after discharge. None of the parameters in both groups decreased to the level indicating a malnutrition status during the follow-up periods. Those parameters were recovered to the preoperative levels within 3 months after discharge and increased continuously. In low rectal surgery, the absorption capacity of the small intestine is almost the same as before surgery. Thus, the recovery rates of these parameters were almost same between the two groups. However, the recovery rates of hemoglobin level showed a different pattern between the two groups. The hemoglobin level in the APR group was recovered to the preoperative level within 3 months of discharge; that in the uLAR group tended to be recovered to their preoperative level after 1 year of discharge. The slow recovery of hemoglobin level in the uLAR group seems to be attributable to the effect of ileostomy. Vitamin B12 and iron deficiencies are common problems in patients with an ileostomy [17, 18]. Although the exact level of vitamin B12 or iron was not analyzed in this study, the decreased absorption capacity of those nutrients could have affected the recovery of hemoglobin in the uLAR group before ileostomy closure.

This study has several limitations owing to its retrospective nature. First, to continuously analyze changes in patients during 3 years, we had to exclude some patients with loss of data owing to irregular follow-up, and this may have caused a selection bias in the results. In addition, the parameters used in our study were selected to monitor the postoperative status of rectal cancer patients; more sensitive and accurate markers reflecting nutritional status, such as the levels of prealbumin, retinol-binding protein, transferrin, and iron could not be measured [13]. In this study, some patients with local or distant recurrences were also excluded to compare the nutritional effect after surgeries, not the outcome of the disease itself.

We also excluded cases that needed a permanent stoma for the treatment of postoperative complications after uLAR because those cases were not suitable for the evaluation of LARS. There was no prophylactic procedure for LARS before ileostomy closure. After ileostomy closure, nutritional counseling or medical supports were provided to the patients who complained about frequent defecation or urgency over 10 times per day. The biofeedback therapy was recommended to the patient who showed intractable low anterior syndromes that persisted at least 1 year after ileostomy closure. The biofeedback therapy in our institute included coordination training, sensory training, and strength training and was performed once weekly for 10 consecutive weeks. Five (27.8%) patients in the uLAR groups had underwent biofeedback therapy and two of them reported improved symptoms. One patient did not show any change after therapy, and the other two patients refused to continue the biofeedback therapy. We could not include the results after these measures for patients in this study, because of the lack of medical records that came from the limitation of the retrospective study.

A study including those cases might find a relationship between nutritional and oncological status after uLAR and APR. Additionally, in order to observe when patients in the uLAR group completely restored their preoperative weight and when a plateau of the nutritional parameters was formed in the two groups, it seems necessary to conduct a study with a follow-up period of 3 years or more. According to the results of other studies, patients who underwent uLAR tended to adapt to their defecation problems over time, and if the patients are free of cancer after 5 years, the presence of an abdominal stoma impairs the QoL to a greater extent than do LARS-related problems [19, 20]. It is also unclear that weight gain of the APR group was not associated with increased food intake with limited activities and mood disorders after APR. Therefore, a more long-term investigation of nutritional status and QoL including the patients' defecation and eating habits, with a questionnaire survey conducted concurrently, will better clarify the association between LARS after uLAR and the nutritional status over time.

## 5. Conclusions

In conclusion, although the capacity of recovering the preoperative body weight in patients who underwent uLAR was not low to the extent of aggravating the patients' nutritional status, it was slower than that in patients who underwent APR. LARS might be one of the primary causes of this difference, but more studies are required to clarify this relationship. Even so, the results of this study might indirectly reflect the low anterior syndrome effect of uLAR and help colorectal surgeons in selecting better surgical methods and in better counseling patients with very low-lying rectal cancer.

## Figures and Tables

**Table 1:** Demographics and preoperative data.

	Total (*n* = 35)	APR (*n* = 17)	uLAR (*n* = 18)	*p* value
Age at operation, years	57.9 ± 10.3	56.6 ± 10.9	59.0 ± 9.8	0.495
Sex: male, *n* (%)	19 (54.3)	11 (64.7)	8 (44.4)	0.229
BMI, kg/m^2^	23.3 ± 2.3	23.7 ± 2.5	23.0 ± 2.3	0.423
Body weight, kg	60.6 ± 7.7	61.0 ± 7.8	60.2 ± 7.7	0.572
Protein, g/dL	7 ± 0.59	7.0 ± 0.7	7.0 ± 0.48	0.912
Albumin, g/dL	4.1 ± 0.4	4.1 ± 0.5	4.2 ± 0.29	0.458
Hemoglobin, g/dL	12.3 ± 1.0	12.0 ± 1.1	12.6 ± 0.9	0.07
Lymphocytes, mm^3^	1183 ± 425	1206 ± 501	1162 ± 352	0.766
PCRT, *n* (%)	32 (91.4)	16 (94.1)	16 (88.9)	0.581
Tumor location from AV, cm	2.2 ± 0.77	1.62 ± 0.63	2.72 ± 0.43	<0.001
Clinical T category, *n* (%)				0.684
1-2	9 (25.7)	5 (29.4)	4 (22.2)	
3-4	26 (74.3)	12 (70.6)	14 (77.8)	
Clinical N category, *n* (%)				0.318
0	8 (22.9)	3 (17.6)	5 (27.8)	
1	11 (31.4)	4 (23.5)	7 (38.9)	
2	16 (45.7)	10 (58.8)	6 (33.3)	

APR: abdominoperineal resection; uLAR: ultralow anterior resection; BMI: body mass index; PCRT: preoperative chemoradiation therapy; AV: anal verge.

**Table 2:** Operative and pathologic results, n (%).

	Total (*n* = 35)	APR (*n* = 17)	uLAR (*n* = 18)	*p* value
Interval from PCRT to operation, weeks^a^	7.0 ± 0.91	6.85 ± 0.85	7.12 ± 0.97	0.46
Surgical approach				0.086
Open	29 (82.9)	16 (94.1)	13 (72.2)	
Minimally invasive	6 (17.1)	1 (5.9)	5 (27.8)	
Ligation of IMA (high)	14 (55.6)	7 (53.8)	8 (57.1)	0.863
Pathologic T category				0.779
1-2	11 (32.4)	4 (23.5)	7 (38.9)	
3-4	25 (68.6)	13 (76.5)	11 (61.1)	
Pathologic N category				0.486
0	30 (85.6)	14 (82.3)	16 (88.8)	
1	4 (11.5)	3 (17.7)	1 (5.6)	
2	1 (2.9)	0	1 (5.6)	
Adjuvant chemotherapy	33 (94.3)	17 (100)	16 (88.9)	0.157
Adjuvant radiation therapy	2 (8)	1 (8.3)	1 (7.7)	0.953

^a^Data from 32 patients who underwent preoperative chemoradiation therapy. APR: abdominoperineal resection; uLAR: ultralow anterior resection; PCRT: preoperative chemoradiation therapy; IMA: inferior mesenteric artery.

**Table 3:** Risk factors of weight loss at 3 years after the operation compared with the preoperative body weight.

Parameters	Univariate analysis		Multivariate analysis^a^	
	*β*-Coefficient (95% CI)	*p* value	*β*-Coefficient (95% CI)	*p* value
Age	1.04 (0.97–1.11)	0.31		
Sex: female	1.16 (0.30–4.40)	0.831		
PCRT^a^	0.39 (0.03-4.74)	0.46		
Ligation of IMA (high)	3.03 (0.75–12.21)	0.12		
Adjuvant chemotherapy	0.83 (0.05–14.48)	0.9		
Adjuvant radiation therapy	1.20 (0.07–20.85)	0.9		
Clinical T category	1.07 (0.23–4.92)	0.93		
Type of surgery (uLAR)	12.13 (2.41–61.20)	0.003	10.147 (1.01–101.8)	0.049
Length from AV	3.57 (1.22–10.42)	0.02	1.2 (0.25–5.61)	0.835

^a^ *R* ^2^ = 0.459. PCRT: preoperative chemoradiation therapy; IMA: inferior mesenteric artery; uLAR: ultralow anterior resection; AV: anal verge.

## Data Availability

The data used to support the findings of this study are available from the corresponding author upon request.
